# The prevalence and pattern of deciduous molar hypomineralization and molar-incisor hypomineralization in children from a suburban population in Nigeria

**DOI:** 10.1186/s12903-015-0059-x

**Published:** 2015-06-30

**Authors:** Oluwaseyi Dada Temilola, Morenike Oluwatoyin Folayan, Titus Oyedele

**Affiliations:** Obafemi Awolowo University Teaching Hospitals Complex, Ile-Ife, Nigeria; Obafemi Awolowo University, Ile-Ife, Nigeria; Oral Habit Study Group, Ile-Ife, Nigeria

**Keywords:** Prevalence, MIH, DMH, Nigeria, Age, Sex, Socioeconomic

## Abstract

**Background:**

Molar Incisor Hypoplasia (MIH) and Deciduous Molar Hypoplasia (DMH) have significant impact on the quality of life of affected individuals. The objective of the study was to determine the prevalence, pattern and clinical presentation of MIH and DMH in children resident in Ile-Ife, Nigeria, and their association with sex and socioeconomic status of the children.

**Methods:**

Information on age, sex and socioeconomic status was collected from 563 children aged 3 to 5 years and 8 to 10 years using a structured questionnaire through a household survey. Clinical examination was conducted to assess for the presence of DMH and MIH. The prevalence of DMH and MIH were determined. Tests of association between sex, socioeconomic status, prevalence, and pattern of presentation of both DMH and MIH were conducted using Pearson’s Chi-squared test Fisher’s exact test.

**Result:**

Fifteen (4.6 %) of the 327 children aged 3 to 5 years and 23 (9.7 %) of the 237 children aged 8 to 10 years had DMH and MIH respectively. There were no significant association between DMH, sex (*p* = 0.49) and socioeconomic status (*p* = 0.32). There were also no significant association between MIH, sex (*p* = 0.31) and socioeconomic status (*p* = 0.41). MIH/DMH co-morbidity was observed in eight (34.8 %) of the 23 children with MIH. The mandible and maxilla were affected equally. Antimere was not observed.

**Conclusion:**

The prevalence of DMH and the prevalence of MIH in the study population were high. DMH and MIH were not associated with sex and socioeconomic status. There was no specific pattern identified in the presentation of DMH and MIH. The prevalence of DMH/MIH co-morbidity is also high. Patients with DMH should be screened for MIH.

## Background

Developmental defects of dental enamel are common in both deciduous and permanent dentitions. These defects include hypomineralization of the enamel [1, 2]. Hypomineralisation is defined as a qualitative defect of the enamel caused by a disturbance during initial calcification and/or during maturation [1, 3]. Hypomineralization is an important risk factor for caries in the primary dentition [4], and caries progresses rapidly in the permanent teeth which is hypomineralized [5].

One form of tooth hypomineralization is Molar incisor hypomineralisation (MIH) defined as the developmentally-derived dental defect that involves hypomineralisation of 1 to 4 first permanent molars (FPM) and frequently associated with similarly affected permanent incisors [3, 6]. The prevalence varies considerably throughout the world ranging from 2.5–40.2 % [7–13]. The lesion presents as demarcated enamel opacities of different colour in the affected teeth. Affected teeth occasionally undergo post-eruptive breakdown due to soft and porous enamel, resulting in atypical cavities or even to complete coronal distortion [3].

MIH-like defects have also been observed on second primary molars and permanent cuspids [3]. These MIH-like defects in the primary molars are now described as Deciduous Molar Hypomineralization (DMH) [4, 7]. DMH might be used as a predictor for MIH because of overlaps in the development process of the second primary molars and the first permanent molars . The development of the second primary molars starts at around the same time as the development of the first permanent molars and permanent incisors, but the maturation of the permanent teeth occurs more slowly [14, 15]. If these teeth are exposed to insults during this overlapping period, hypomineralization might occur in the primary as well as in the permanent dentition [16].

Children with MIH need more dental treatments and – probably as a consequence – are generally more fearful than their peers [5]. Therefore, it is important to diagnose MIH as early as possible to reduce the vulnerability of the MIH-affected molars by focusing on their restorative and preventive needs. The diagnosis of MIH and DMH are both clinical. Initially the tooth develops normally but the enamel begins to show areas of weakness and breakage. The affected tooth appears with thinned enamel surface and postoperative demarcated opacities [17]. Radiographic evaluation of the affected tooth may show normal morphology of the crown but reduced enamel opacity that may approach that of the dentine.

Very few studies have reported the relationship between DMH and MIH [13, 18]. The clinical importance of MIH and the possibility of DMH serving as a predictor of MIH make it important to conduct more studies to determine the prevalence and associations between the two lesions. Also, the increased risk of caries, hypersensitivity and aesthetic concerns for patients with MIH [19] and the negative impact of MIH on the quality of life of children with the lesion make it important to understand further patterns of presentation of MIH and DMH in various populations. Furthermore, the high prevalence of enamel hypoplasia in the study population [20] makes it imperative to further explore the prevalence of other enamel defects in the study population such as MIH and DMH, which may also increase the risk for caries just like enamel hypoplasia [21]. The aim of this study therefore, was to determine the prevalence, and pattern of presentation of MIH and DMH in the primary dentition and mix dentition of children resident in Ile-Ife, a suburban region of Nigeria. It also examined the association between the diagnosis of MIH and DMH, sex and socioeconomic status of the children.

## Methods

This cross-sectional study was conducted in Ile-Ife Central Local Government Area (LGA). It was part of a larger study of which the study population, sample size, sampling techniques had been described in great details in a prior study [20]. Data was collected through a household survey administered to 993 children aged 4 months to 12 years old. Children excluded from the study were those who had a medical condition or syndrome associated with tooth anomalies, those who had cleft palate, and those with a history of diseases that could increase the risk for developing dental anomalies, such as maternal syphilis.

For this study, a subset of children aged 3 to 5 years and 8 to 10 years of age were analyzed. To determine prevalence of MIH in the study population, it was estimated that the proportion of children with MIH was 40 %, using the highest reported prevalence from various studies reporting the prevalence of MIH [22, 23]. The appropriate sample size for such a study with a 10 % attrition rate using the statistical formula proposed by Araoye [24] was 405 children. We however had a study population of 563 children for this study.

### Data collection tool

Data collection was through the use of an interviewer administered questionnaire. A dentist conversant with normal and pathological dental features and who had been engaged in a similar household dental survey in the same LGA, was engaged as a field worker for the study. Data collected included information on the child’s socio-demographic characteristics (age, sex, and socioeconomic status). Age was defined as age at last birthday. Socioeconomic status for the purpose of this study was obtained through a multiple item scoring index [25] used in prior studies in Nigeria [26, 27]. The status designation combines the mother’s level of education with the occupation of the father; each child was allocated to a social class I to V, with social class V being lowest. Each child’s social class was classified as Class I (upper class), class II (upper middle class), class III (middle class), class IV (lower middle class) and class V (lower class).

### Clinical examination

All children eligible to participate in the study had an oral examination. The examinations were conducted under natural light, with the children sitting on a chair. The teeth were examined wet after debris had been removed by use of a piece of gauze using sterile dental mirrors and probes. The dental mirror was used to further provide illumination of the tooth surfaces through reflection of light and sun rays. Each tooth that had fully emerged into the mouth was screened for MIH or DMH using the criteria described by Kemoli et al. [8]. The coronal part of the second primary molars, permanent first molars and permanent incisors were thoroughly examined for evidence of enamel hypomineralization. A tooth was considered to have MIH or DMH when there was a demarcated opacities of about 2 mm associated with or without post-operative defects of deficiency in the enamel, large and extensive restorations on any of these teeth and suspected to be a result of hypomineralization. A diagnosis of MIH or DMH was only made when at least one molar was affected, with or without the involvement of the incisors [28].

### Standardization of examiner

An intra-examiner reliability test was done to calibrate the principal investigator on consistency of diagnosis for hypomineralization. The test was done by examining photographs of Hypomineralized molars and incisors. The scoring of the pictures identified correctly was recorded and repeated twice at an interval of one week. The intra-examiner reliability score was 0.90.

### Data analysis

The ages of the study participants were divided into two categories for data analysis: 3–5 years and 8–10 years. The socioeconomic status of the children was also re-categorized into three classes: social classes I and II, high socioeconomic status; social class III, middle socioeconomic status; and social class IV and V, low socioeconomic status. Descriptive analysis was conducted to determine the prevalence of MIH and DMH in the study environment. Tests of association between dependent variables (presence of DMH and MIH) and the independent variables (socioeconomic status and sex) were conducted using where appropriate, the Pearson’s Chi-squared test Fisher’s exact test. Statistical analysis was done with Intercooled STATA (release 12) for windows. Simple proportions were computed. Statistical significance was inferred at p < 0.05.

### Ethical consideration

Ethical approval was obtained from the Obafemi Awolowo University Teaching Hospital Complex Ile-Ife. Approval for community entry was obtained from the LGA office. Written informed consent was obtained from a parent or legal guardian of each study participant prior to enrollment. Information on the socio-demographic profile of the children was obtained from either of the consenting parent or legal guardian and assent for children 8 years and above.

## Results

The 563 study participants were stratified into two groups for data analysis. These were 327 (58.1 %) 3–5 years old children who were examined for DMH and the 236 (41.9 %) 8–10 years children who were examined for MIH.

### Prevalence and pattern of DMH

Fifteen (4.6 %) of the 327 3–5 years old children had DMH. Table 1 provides a summary of the socio-demographic profile of children diagnosed with and without DMH. Seven (46.7 %) of the 15 children were from low socioeconomic class, seven (46.7 %) were from middle socioeconomic class while one (6.6 %) was from high socioeconomic class. There was no statistically significant difference in the socio-economic class (*p* = 0.32) of children with DMH. Also, nine (60 %) of the children were females. There was no statistically significant difference in the sex distribution (*p* = 0.49) of children with DMH.

**Table 1:** Prevalence and pattern of distribution of DMH by socio-demographic profile

Variables	Presence of DMH *n* =15	Absence of DMH *n* =312	Total *N* = 327	*P* value
Socioeconomic status				
High socioeconomic class	1 (6.6 %)	71 (22.7 %)	72 (22.0 %)	0.32
Middle socioeconomic class	7 (46.7 %)	102 (32.7 %)	109 (33.3 %)	
Low socioeconomic class	7 (46.7 %)	139 (44.6 %)	146 (44.7 %)	
Sex				
Male	6 (40.0 %)	153 (49.0 %)	159 (48.6 %)	0.49
Female	9 (60.0 %)	159 (51.0 %)	168 (51.4 %)	

Forty five of 1305 erupted second primary molars (3.4 %) were affected with DMH. Of these, 22 (48.9 %) were present in the mandible and 23 (51.1 %) were present in the maxilla. Also, 21 of the 45 affected teeth (46.7 %) were present on the right quadrant while 24 teeth (53.3 %) were present in the left quadrant.

### Prevalence and Pattern of MIH

Twenty three (9.7 %) of the 236 children aged 8–10 years had MIH. Table 2 rovides a summary of the socio-demographic profile of children diagnosed with and without MIH. Seven (30.4 %) of the 23 children with MIH were from low socioeconomic class, 10 (43.5 %) were from the middle socioeconomic class while six (26.1 %) were from high socioeconomic class. There was no statistically significant difference in the socio-economic class (*p* = 0.41) of children with MIH. Also, 14 of the 23 children (60.9 %) were females There was no statistically significant difference (*p* = 0.31) in the sex distribution of children with MIH.

**Table 2:** Prevalence and pattern of distribution of MIH by socio-demographic profile

Variables	Presence of MIH *n* =23	Absence of MIH *n* =213	Total *N* = 236	*P* value
Socioeconomic status				
High socioeconomic class	6 (26.1 %)	48 (22.5 %)	54 (22.9 %)	0.41
Middle socioeconomic class	10 (43.5 %)	70 (32.9 %)	80 (33.9 %)	
Low socioeconomic class	7 (30.4 %)	95 (44.6 %)	102 (43.2 %)	
Sex				
Male	9 (39.1 %)	107 (50.2 %)	116 (49.2 %)	0.31
Female	14 (60.9 %)	106 (49.8 %)	120 (50.8 %)	

Also, 53 of the 937 erupted first permanent molars (5.7 %) and 22 of the 923 fully erupted first permanent incisors (2.4 %) teeth were affected with MIH. Of the 75 affected teeth 30 (40 %) teeth were in the mandible and 45 (60 %) teeth were present in the maxilla. Also, 35 of the 75 affected teeth (46.7 %) were on the right quadrant while 40 affected teeth (53.3 %) were on the left quadrant.

Table 3 shows the mean number of affected permanent incisors, according to the number of affected permanent first molars. Nine of the 23 children (39.1 %) had first molars affected by MIH with no concurrent involvement of the incisor. The highest mean number of incisor involvement was found in children who had all the four permanent first molars involved (2.2 ± 1.1). The mean number of teeth affected by MIH per child was 3.5 (±1.8). Most of the children (65.2 %) had more than two teeth affected.

**Table 3:** Mean number and standard deviation of affected permanent incisors according to the number of affected permanent first molars

Number of permanent first molar	Mean number of affected incisor
1	2.0 (±0.0)
2	2.0 (±1.2)
3	1.3 (±0.6)
4	2.2 (±1.1)

Eight (3.4 %) of the 236 children examined for MIH had MIH/DMH co-morbidity. Thus, 34.8 % of the children with MIH had MIH/DMH co-morbidity.

## Discussion

This study makes a unique contribution to the growing literature on the epidemiology of MIH and DMH. A number of recent studies on MIH have focused on its possible determinants [29, 30] so as to help facilitate early diagnosis and prompt management of MIH. Because the second primary molars erupt 4 years earlier than the first permanent molars, DMH might be a clinically useful predictor for MIH and hence the need for more research into the relationship between DMH and MIH. Currently, there is paucity of information on the subject.

The finding of this study showed that a significant proportion of children in the study environment had DMH, MIH and MIH/DMH co-morbidity. These lesions did not have sex and socio-economic status predilection. The mandible and maxilla were affected equally. Antimere was not observed. Most of the children with MIH, had more than two teeth affected, with no trend observed between the number of molars affected by MIH, and the number of incisors affected.

The prevalence of DMH of 4.6 % reported in our study is close to the 4.9 % reported in children in the Netherlands [11]. However, our reported prevalence of MIH is higher than the 5.9 % prevalence reported in Germany [31] and lower than the 13.7 % prevalence report in Kenya [8] and the 17.6 % prevalence reported in a prior study conducted in Nigeria [32]. The studies on MIH conducted in Kenya and Nigeria were school based studies. School based oral health studies have limitations for its generalizability for children in Nigeria because of the large number of out-of-school children: about 40 % of children of primary-school age and 60 % of children of secondary-school age are out of school. The country also accounts for 47 % of the world’s out-of-school population [33].

The high proportion of primary and permanent molars affected by DMH and MIH is a cause for concern. MIH and DMH have been associated with increased risk for caries [34]. Unfortunately, management of caries is often delayed [35], with quality of life of children with caries and caries sequelea significantly affected [36]. Conducting school oral health screening programmes may indeed enhance early diagnosis of the lesion of a large number of cases as the results of the study by Oyedele et al. [19] showed. Prompt diagnosis does not however infer prompt treatment as attendance at dental clinics following referrals from school oral health screening programmes is also poor in the study environment [37]. This implies the need for augmentation of school based oral health screening programmes with school based treatment programmes, as well as community based treatment programmes so as to facilitate access of out-of-school children to oral health care.

The high MIH/DMH co-morbidity observed in this study further validates the findings by Elfrink et al. [13]. This finding also implies that a DMH/MIH may have a genetic predisposition or the lesion results from a chronic or frequent recurrent disease that happened within a particular time span rather than only one risk factor affecting first, the second primary molars and, later, the first permanent molars. DMH might therefore serve as a good and useful clinical indicator for MIH.

The study found no sex predilection for DMH and MIH; a similar findings that had been reported in prior studies [38, 39]. The study also observed no association between DMH, MIH and the socioeconomic status of the child thereby confirming the observation made by Oyedele et al. [19] in the study environment. This observation differs from prior observation on the relationship between prevalence of enamel defects and socioeconomic status [38, 40, 41]. Like Oyedele et al. [19] observed, the non-association between DMH, MIH and socioeconomic may serve as a distinguishing feature between enamel hypoplasia and DMH/MIH in the study environment.

This study has a methodological strength: it conducted a household survey, thus increasing the chances of including both in- and out-of-school children of all age groups and socioeconomic class in the study population. The result is therefore generalization to the study population. The study can however not be generalized to Nigeria as a country as the study was only conducted in one of the 774 LGAs in Nigeria. It would be important to scale up this study using a nationally representative sample so as to generate information on the national prevalence of DMH and MIH. Also, what we notice in this study is an overlap of the occurrence of DMH and MIH [42]. A prospective study on the association between DMH and MIH would be able to provide definitive findings on the association between DMH and MIH.

## Conclusion

This study showed that DMH and MIH were not associated with sex and socioeconomic status. There was no specific pattern identified in the presentation of DMH and MIH. The high prevalence of DMH/MIH co-morbidity is a justification for screening children with DMH for MIH.

## Abbreviations

DMH: Deciduous molar hypomineralisation
FPM: First permanent molar
LGA: Local government area
MIH: Molar incisor hypomineralization
